# The Phenolic Content of *Pistacia lentiscus* Leaf Extract and Its Antioxidant and Antidiabetic Properties

**DOI:** 10.1155/2024/1998870

**Published:** 2024-02-07

**Authors:** Hamza Bouakline, Saliha Bouknana, Mohammed Merzouki, Imane Ziani, Allal Challioui, Mohamed Bnouham, Abdesselam Tahani, Ali EL Bachiri

**Affiliations:** ^1^Physical Chemistry of Natural Substances and Process Team, Laboratory of Applied Chemistry and Environment, Faculty of Sciences, University Mohamed Premier, Oujda, Morocco; ^2^Laboratory of Bioresources, Biotechnology, Ethnopharmacology and Health, Faculty of Sciences, University Mohamed Premier, Oujda, Morocco; ^3^Laboratory of Applied Chemistry and Environment (LCAE-ECOMP), Faculty of Sciences, University Mohamed Premier, Oujda, Morocco

## Abstract

The aims of this study were to determine the polyphenolic profile, to estimate the total phenolic and flavonoid contents, and to evaluate the antioxidant and antidiabetic activities of the extract of *Pistacia lentiscus* leaves, and the hydroacetonic mixture was employed as an alternative for common solvents in the extraction process. In order to explain the antidiabetic activity, molecular docking has been performed on the main constituents of the leaf extract. The characterization of the extract has been performed by high-performance liquid chromatography (HPLC) leading to the detection of 20 compounds of which gallic acid, ellagic acid, catechin, kaempferol, and quercetin 3-glucoside were identified using authentic standards. The total phenolic and flavonoid contents, assessed using the Folin–Ciocalteu and quercetin methods, were 394.5 ± 0.08 mg gallic acid equivalent/g dry extract (mg GAE/g DE) and 101.2 ± 0.095 mg quercetin equivalent/g dry extract (mg QE/g DE), respectively. On the other hand, the antioxidant activity of leaf extract, quantified by determining the ability to neutralize the free radical DPPH and *β*-carotene/linoleate model system, reached the values of 0.0027 ± 0.002 mg/mL and 0.128 ± 0.04 mg/mL, respectively. Regarding the antidiabetic activity, based on the inhibition of pancreatic *α*-amylase activity, a significant inhibition of about 68.20% with an IC_50_ value of 0.266 mg/mL had been observed. This finding is consistent with the molecular docking study of the main phenolic compounds of the extracts, where a remarkable binding affinity against *α*-amylase was observed, with values of −7.631 (kcal/mol), −6.818 (kcal/mol), and −5.517 (kcal/mol) for the major compounds catechin, quercetin-3-glucoside, and gallic acid, respectively.

## 1. Introduction

*Pistacia lentiscus* L., a wild-growing species classified within the family of Anacardiaceae, is one of more than 4,200 varieties, including 600 plants with medicinal and aromatic properties [1, 2]. Several studies into the ethnobotany of the plant under study have been carried out, as previously reported in various works [3–5]. Recently, especially in the new century, products of natural origin, such as essential oils and plant extracts, have attracted the attention of researchers and become popular with consumers. Their use as an alternative to synthetic products [6, 7] appears to reduce production costs and avoid many negative side-effects on human health. Many scientists and researchers are focusing on emerging natural materials such as natural plant extracts, in particular *Pistacia lentiscus* leaf extract, with the aim of creating new horizons for scientific research on the one hand [8] and, on the other, to investigate potential uses for these natural compounds in the pharmaceutical sector, particularly as antioxidant agents or inhibitors of enzymes such as *α*-amylase and *α*-glucosidase. The aim of this study is also to justify the traditional use of this plant in the treatment of various illnesses, including diabetes, as observed in many Third World countries [9, 10].

The chemical profiles of the essential oil and phenolic compounds extracted from the leaves of *P. lentiscus* were detailed in the study conducted by Amhamdi et al. The researchers identified more than 35 components in the essential oil, with major compounds such as myrcene, limonene, *β*-gurjunene, and germacrene. In addition, *α*-pinene and *β*-pinene were noted as abundant compounds [11]. The extracts contain more than 40 phenolic compounds, classified into three main classes of secondary metabolites [12]. The plant has also been extensively studied for a variety of biological activities, which have been reported to possess antifungal [13], antibacterial [14, 15], antioxidant [16–19], antimalarial, and antiviral activities [20], as well as anti-inflammatory [21], analgesic, and antithermogenic activities [22, 23].

Concerning the antidiabetic activity, research scientists have demonstrated that the extract exhibited significant inhibition of pancreatic amylase at the low doses ranging between 60 and 100 *μ*g/mL [10]. Enzymes such as oxidases participate in various metabolic processes, and their connection to the antidiabetic activity may be indirect. For instance, certain studies propose that antioxidant enzymes, including oxidases, might contribute to alleviating oxidative stress associated with diabetes [9, 24]. Increased oxidative stress is associated with the onset and advancement of diabetes. In addition, some plant-derived compounds with antioxidant properties that could influence the oxidase activity have been studied for their potential antidiabetic effects [25]. Furthermore, some researchers examined the antioxidant efficacy of the extract obtained from the leaves of *P. lentiscus* and its primary components, both experimentally and theoretically, in order to detect the specificity of the binding between the main compounds identified in the *Pistacia lentiscus* leaf extract [17, 26].

The essential oil and extract of this plant are effective against Gram-negative bacteria, such as *Pseudomonas aeruginosa* and *Escherichia coli*, as well as Gram-positive bacteria including *Bacillus subtilis* and *Listeria monocytogenes* [27]. Furthermore, various phytochemical studies have shown the considerable existence of some polyphenolic compounds such as flavonoids [28] and tannoids and also the appearance of sterols, saponins, and triterpenes [29, 30]. Among the reasons that allowed the development of the extract of *Pistacia lentiscus* leaf, either as an antioxidant agent or as an inhibitor of the enzymes responsible for diabetes to act as a trophic agent on the acinar tissue, to augment the production of enzymes of the cultured islets [31], there is first of all the absence of symptoms of toxicity, and then the abundance and wild growth of this species are additional aspects that qualify for a detailed study. Enhanced by the limited research, even those that are available are focused on the experimental aspect and neglect the theoretical aspects. At this point, in our study, the focus was experimental, examining the performance of the extract and three of its main compounds. These compounds belong to two different families of polyphenols, the flavonoid family and the phenolic acid family. The aim was to determine the effect of the chemical formula rich in polyhydroxyl blocks and its relationship to inhibitory activity. Theoretically, the calculations approved the result obtained. On the toxicity side, Remila [32] proved that although the extract concentration reached 100 *μ*g/mL, no toxicity was observed, in addition to the antioxidant aspect expressed, to the fact that these extracts contain high levels of vitamin C, as reported earlier by Hadini [33] since the amount of vitamin C has reached approximately 22 mg ascorbic acid per 1 g of dried matter of the extract. Hence, the main aims of this research involve elucidating the chemical composition of the hydroacetonic extract obtained from *Pistacia lentiscus* leaves and quantifying its total phenolic and flavonoid contents. Furthermore, the investigation aims to assess the antioxidant potential and the antidiabetic properties of the extract. Another integral objective is to conduct molecular docking studies of the predominant phenolic compounds. This work is considered one of the first of its kind on this plant combining the antidiabetic activity of its extract and its theoretical explanation.

## 2. Materials and Methods

### 2.1. Plant and Material

**Figure 1:**
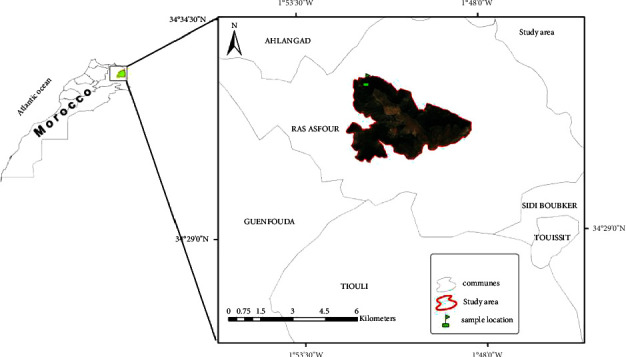
The sampling collection.

The *Pistacia lentiscus* leaves used in this research were collected from the northeastern part of Morocco (altitude: 836 m, 34° 32′ 52.968″N 1° 51′ 25.301″W) in March 2021 (Figure 1). All the reagents, solvents, and standards were purchased from Sigma-Aldrich.

### 2.2. Procedure of Extraction

An amount of 5 g of grinded leaves was mixed with 50 mL of a hydroacetonic mixture at a ratio of (80/20), respectively, for 12 h under mechanical stirring. The resulting extract was stocked at 4°C for 24 h, followed by vacuum filtration, evaporation under vacuum, and storage until it was needed for the different analyses [34].

### 2.3. HPLC Analysis

*Pistacia lentiscus* hydroacetonic extract samples were prepared in MeOH using the rapport of 10 mg in 1 mL, and then filtration with a suitable accessory was done (filter of 0.45 *μ*m pore size), following a direct injection of a volume 10 *μ*L into high-performance liquid chromatography (HPLC-DAD) (Waters Alliance™ e2695 XC HPLC System), which is integrated with a diode array detector (DAD) and also an apolar column C18 (5 *µ*m, 250 mm × 4.6 mm). To start the analysis procedure [35], the polyphenolic compound detection was accomplished by the following steps and the gradient mode was used as an elution program with a flow rate of 0.7 mL/min. Water 0.75% and the rest of acetic acid were used as the first eluent (A) on the one hand and the methanol as the second eluent (B) on the other; the elution operation was performed according to the gradient in Table 1, as described by Khodir et al., with some modifications [36].

### 2.4. Determination of Total Phenol Content

The procedure for determining total phenols was executed using the Folin–Ciocalteu method. In each test tube, a volume of 1500 *μ*L of sodium carbonate solution (20%) was added over to 1000 *μ*L of distilled water and 0.5 mL of Folin–Ciocalteu phenolic reagent (10%) as well as 0.1 mL of the samples to be tested. The absorbance of the samples was measured at 760 nm using a T80+ UV-Vis spectrophotometer, after plotting gallic acid as a standard curve with the following concentrations: 0.01, 0.02, 0.03, 0.04, 0.05, and 0.1 mg/ml. The results were expressed as mg of gallic acid equivalent/gram of dry extract (mg GAE/g DE) [10].

### 2.5. Determination of Flavonoid Content

The flavonoids were measured spectrophotometrically by using colorimetric methods, by adding 500 *μ*L of a solution of 133 mg of AlCl_3_ ‏along with 400 mg of CH_3_CO_2_Na dissolved in 100 mL of distilled water to 1 mL of the extract samples (500 *μ*g/mL), followed by the recording of the absorbance values at 430 nm. The plotting of standard curve of quercetin with the following concentrations, 0.01, 0.02, 0.03, 0.04, 0.05, and 0.1 mg/ml, led to the following: mg quercetin equivalent/gram of dry extract (mg QE/g DE) [10].

### 2.6. In Vitro Antioxidant Activity

#### 2.6.1. DPPH Free Radical Scavenging Assay

The antioxidant activity of extracts is tested by using the capacity of antioxidants to scavenge the free radical 2,2-diphenyl-1-picrylhydrazyl. After preparing a DPPH solution in methanol 0.004%, 2.4 mL was then mixed with 0.6 mL of a series of extracts of varying concentrations, and all the absorbance changes were measured at 517 nm. The values obtained from absorbance measurement are used for the determination of the IC_50_ values, which indicate the antioxidant capacity relative to the values of vitamin C (ascorbic acid) used as a standard [37] according to the following formula:

(1) $$\begin{array}{c} I\%=\frac{A0-\text{AE}}{A0\;}\times 100, \end{array}$$

where *A*0 = the absorbance of the control sample and AE = the absorbance of the sample.

#### 2.6.2. *β*-Carotene Bleaching Model System (BBC)

Based on the concept that the presence of natural antioxidant compounds could neutralize the linoleate free radical, therefore preventing the bleaching of *β*-carotene, the BBC assay for the *Pistacia lentiscus* leaf extract was determined, following the protocol applied by Bekkouch et al. [38], with few modifications. Briefly, the *β*-carotene/linoleic acid emulsion was prepared by dissolving 2 mg in 10 mL of chloroform, followed by a quick addition of 20 mg of linoleic acid and 200 mg of the Tween-80. Next, the chloroform evaporated, and 100 mL of the bidistilled water was added. For the test execution, 2.5 mL of the emulsion solution formed was blended with 0.5 mL of the hydroacetonic extracts or butylated hydroxyanisole (BHT) solution at various concentrations. The absorbance was determined using a UV-Vis spectrophotometer at 470 nm at *t* = 0 and at *t* = 120 min after 2 h incubation at 50°C in a water bath. The inhibition efficiency was calculated according to the following formula:

(2) $$\begin{array}{c} I\%=\frac{\text{AE }\left(120\right)\;–\text{ AC }\left(120\right)}{\text{AE }\left(0\right)\;–\text{ AC }\left(120\right)\;}\times 100, \end{array}$$

where AE (0) and AE (120) sample absorbances were at 0 min and 120 min, respectively, and AC (0) and AC (120) control absorbances were at 0 min and 120 min, respectively.

### 2.7. *In Vitro* Antidiabetic Activity

The *in vitro* antidiabetic activity based on the inhibition of pancreatic *α*-amylase activity was performed on the hydroacetonic extract according to the method of Bouknana et al. [39]. The following concentrations of the extract were tested: 0.05, 0.11, 0.23, and 0.45 mg/ml. In brief, each test tube received 200 *µ*L of extract and 200 *µ*L of *α*-amylase and was incubated at 37°C for 10 minutes. The mixture was then reincubated for 15 minutes at 37°C with 200 *µ*L of 1% starch solution added to each test tube. In order to stop the reaction, 600 *μ*L DNSA reagent (30 g of sodium potassium tartrate tetrahydrate in 20 ml of 2 M NaOH and 40 mL of 96 mM of 3,5-dinitrosalicylic acid solution) was added, as a next step for 8 min, and the mixture was boiled in a boiling water bath. Next, the mixture was cooled at room temperature and diluted with 1 mL of deionized water. On the other hand, a blank with 100% enzyme activity was prepared by replacing the sample volume (200 *μ*L) with the phosphate buffer solution. Blank reaction was equivalently adjusted using samples at each concentration in the absence of blank solution. Acarbose has been used as a positive control sample. All the absorbance measurements were performed at 540 nm.

The inhibition percentage was calculated using the following formula:

(3) $$\begin{array}{c} I\%=\frac{\left(\text{Ac}-\text{Aa}\right)}{\text{Ac}}\times 100, \end{array}$$

where Ac = the absorbance of the control sample and Aa = the absorbance of the sample.

### 2.8. Molecular Docking

The six selected compound structures were obtained from the online PubChem database [40]. The 3D structures and the geometric optimizations with ligand energy minimization were performed using controlled algorithms in Schrodinger 2018-1. Then, the LigPrep module was used by adding hydrogen atoms and eliminating salt and ionization at pH (7 ± 2). Concerning *α*-amylase, molecular docking was achieved from the X-ray crystal structure of human pancreatic *α*-amylase complexing with mini-montbretin A (PDBID: 5 E0F) [40], with a resolution of 1.40 Å. The free *R* value is 0.181, and the working *R* value is about 0.155. The observed *R* value is also 0.156. The protein structure was prepared using the protein preparation wizard (Schrodinger 2018-1), where the water atoms and all the ligands were detached, although the nonpolar hydrogens were fused. The active site binding *α*-amylase with mini-montbretin A was chosen as the gate centers. The dimensions of the central grid box were chosen to include all atoms of the ligand set. The grid box site in *α*-amylase was fixed at −8.29, 21.6, and −18.72 Å (for *x*, *y,* and *z*) using 40, 40, and 40 points (for *x*, *y,* and *z*) and using the standard precision (SP) glide score to predict binding free energy and ligand strain energy to select docked poses; the binding affinity (kcal/mol) is the description of the output docking scores. Energy minimization was performed by default by limiting RMSD to 0.3 Å. Finally, to minimize the structure of the protein, the OPLS3 force field was used [40]. The conformations taken into consideration are those with minimal binding energy. They are converted into a three-dimensional diagram displaying the binding interaction of the ligand with the site of the active residues.

### 2.9. Statistical Analysis

The analyses were carried out in triplicate, and all the results were analyzed using “OriginPro version 2018” software and presented as mean values with standard error.

## 3. Results and Discussion

### 3.1. Qualitative Identification of Chemical Composition of Extracts

**Figure 2:**
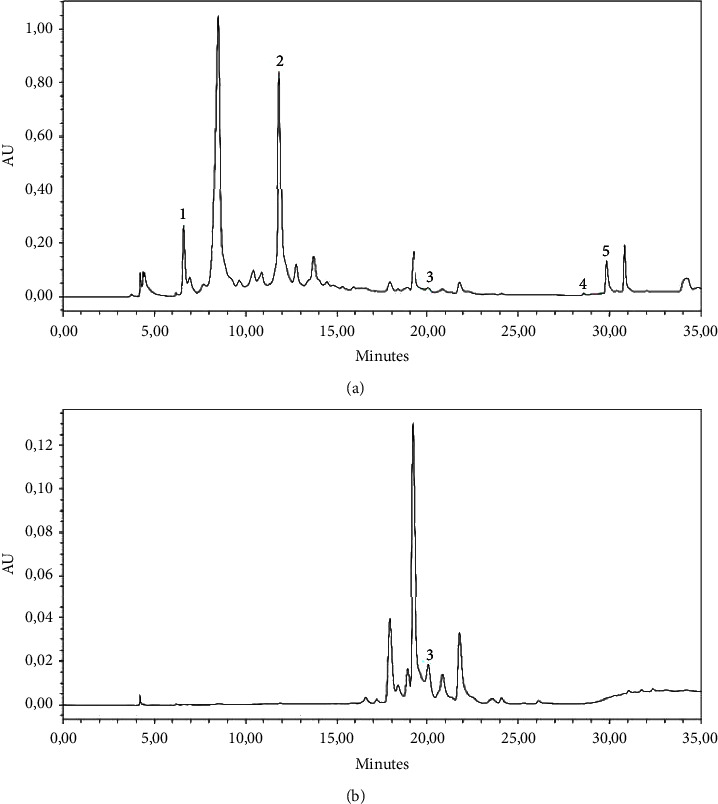
Chromatograms of the *Pistacia lentiscus* leaves at 270 nm and 370 nm.

The chemical profiles of polyphenolic compounds obtained from Pistacia lentiscus leaf extract using HPLC-DAD analysis, with an extraction rate of 39.5%, at two different wavelengths' relative maximum absorbance are presented in Figure 2. The chromatograms revealed the presence of numerous polyphenolic molecules. The identification of some selected phenolics in the extract was carried out using standards, such as gallic acid, catechin, ellagic acid, kaempferol, and quercetin 3-glucoside, by comparing their retention times and *λ*_max_ (Table 2).

The present study supports a previous report on the presence of the gallic acid, p-coumaric acid, and syringic acid as the major phenolic compounds in *Pistacia lentiscus* leaf extracts [41]. Another investigation on *Pistacia lentiscus* leaves carried out by Romani et al. revealed the detection of gallic acid, glucose, quinic acid, myricetin, quercetin, cyanidin 3-O-glucoside, and 3-O-glucoside, with some traces of catechin [12]. Also, our findings are consistent with those reported by Ouahabi et al.; this applies to the leaves of *Pistacia lentiscus* collected in the eastern region of Morocco. It was found that the extract contains many phenolic compounds, such as catechin, quercetin, and p-coumaric acid, along with other notable compounds [42].

### 3.2. Determination of Total Phenol and Flavonoid Contents

The phenolic compound contents estimated from *Pistacia lentiscus* leaf (hydroacetonic) extract are 394.5 ± 0.08 (mg GAE/g DE) for the polyphenols and 101.2 ± 0.095 (mg QE/g DE) for the flavonoids, all of which are listed in Table 3. Concerning the polyphenolic contents in the *Pistacia lentiscus* leaves from the northeastern part of Algeria, values of 517.512 ± 5.53 (mg GAE/g) and 108.67 ± 0.5 (mg QE/g) for the flavonoids were obtained according to Mehenni et al. [10], which are in conformity with the results of our study, and this is primarily due to the geographic proximity of the two harvesting regions. On the other hand, the Bampouli research team came out with 314.88 ± 0.01 (mg GAE/g extract) and 65.18 ± 0.02 (mg QE/g extract) values as a result for polyphenols and flavonoid, respectively [43]. With the aim of comparison, other authors announced that the total phenolic contents of *Pistacia lentiscus* leaf extract from Morocco were 345.95 ± 1.17 (mg GAE/g) [44].

### 3.3. Antioxidant Activity

**Figure 3:**
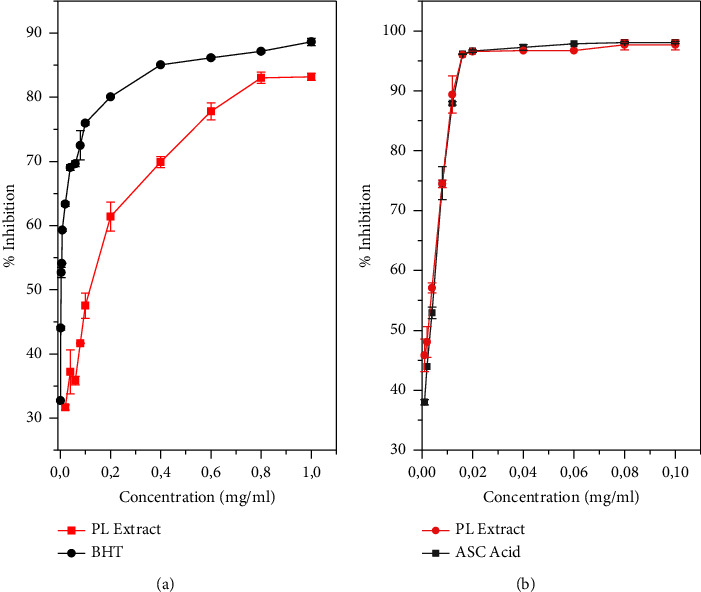
Antioxidant activity of PL extract: *β*-carotene bleaching (a) and DPPH radical scavenging effects (b).

The *in vitro* antioxidant activity performed on the plant extracts has shown a remarkable ability to neutralize free radicals DPPH, IC_50_ = 0.0027 ± 0.002 mg/mL (Figure 3 and Table 3), which is explained by the existence of a high amount of ascorbic acid in extract as Hadini et al. reported [33]. Furthermore, the presence of many different natural antioxidant components such as phenols, flavonoids, and tannins in the plant extract, as already mentioned by the investigation of Bouakline et al. in which the antioxidant activity of the hydroacetonic leaf extract, using the DPPH assays, was tested experimentally and confirmed theoretically by the interaction energy among DPPH and gallic acid, catechin, and quercetin [26]; to support the result of the present study, Yosr et al. [34] announced about this ability of *Pistacia lentiscus* extracts, whereas they found that IC_50_ = 5.7 ± 0.5 *μ*g/mL. Nevertheless, a value of IC_50_ = 0.09 ± 0.00 mg/mL was reported by Barbouchi et al. [44]. However, other different parts of *Pistacia lentiscus* L. extracts have been evaluated for the DPPH antioxidant test, and the leaf extract showed higher ability than that of the other parts [19, 45]. Furthermore, the IC_50_ of *Pistacia lentiscus* leaf extract of our current study was slightly lower than the synthetic antioxidants of the ascorbic acid, with the IC_50_ values of 0.0030 ± 0.003 mg/mL.

The antioxidant activity of the hydroacetonic extracts was evaluated in order to investigate their capacity to inhibit or reduce the bleaching of *β*-carotene as is listed in the table. As a result, the *Pistacia lentiscus* leaf extracts have shown a good performance to inhibit the bleaching of *β*-carotene by scavenging the free radicals formed within the system including the linoleate ones. The concentration 0.128 ± 0.04 mg/mL was recorded to be the value required to give 50% inhibition in the current study, which is different from butylated hydroxyanisole and the pure synthetic standards, IC_50_ = 0.004 ± 0.009 mg/mL; our result was similar to the one of Amessis-Ouchemoukh et al. where IC_50_ found was 0.12 ± 0.00 mg/mL for the methanolic extract of the *Pistacia lentiscus* growing in the suburbs of Bajaia City in Algeria [46]. On the other hand, the different organs of the plant under study were tested as potential natural antioxidants, where the highest activity was observed in leaf extracts with IC_50_ = 0.166 ± 0.001 mg/mL compared to the stems, fruits, and roots with the following values, 0.076 ± 0.001 mg/mL, 0.772 ± 0.043 mg/ml, and 0.370 ± 0.002 mg/mL, respectively [45]. Based on the literature, Ouahabi et al. reported that the *Pistacia lentiscus* leaf extract possesses *β*-carotene bleaching activity comparable to the value obtained in our study, with an IC_50_ value of 0.19 mg/ml [42]. Our obtained results have shown that the high polyphenol content could probably affect the oxidative power of the extract, which is in accordance with the literature search which indicated that higher antioxidant activity was related directly to the high amount of phenolic compounds [46].

### 3.4. Antidiabetic Activity

**Figure 4:**
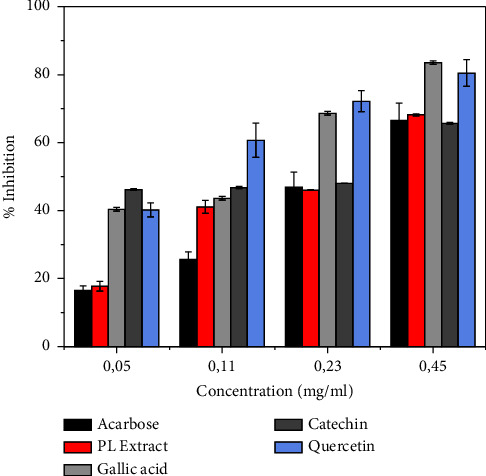
Comparison of the *α*-amylase inhibitory effect of *Pistacia lentiscus* extracts at different concentrations.

The *in vitro* antidiabetic activity performed on *Pistacia lentiscus* leaf extract presented an interesting result concerning the inhibition of *α*-amylase. The inhibition results were 17.75%, 41.11%, 46.64%, and 68.20% for the following concentrations of 0.05 mg/mL, 0.11 mg/mL, 0.23 mg/mL, and 0.45 mg/mL, respectively, with an IC_50_ value of 0.266 ± 0.01 mg/mL, as shown in Figure 4 and Table 3. This result is mainly due to the major polyphenol and flavonoid constituents such as catechin, quercetin, and gallic acid, which were also examined as prospective antidiabetic substances, which have an important IC_50_ represented by, IC_50_ = 0.174 ± 0.001 mg/mL, IC_50_ = 0.058 ± 0.05 mg/mL, and IC_50_ = 0.098 ± 0.001 mg/mL for catechin, quercetin, and gallic acid, respectively, to inhibit the alfa amylase enzyme. Specifically, the catechin, as a compound of the flavonoid family, classified as flavanol subclass, had a significant effect at low concentrations of 0.05 mg/mL, with inhibitory activity greater than other components, as quercetin from the same family listed as the flavonol subgroup. On the same approach, previous works, regarding flavones as flavonoids, were tested, indicating that the position of the hydroxyl group or H group on the ring of flavonoids along with their molecular volume had a significant effect on the inhibitory activity [47, 48]. However, some phenomena were observed in the inhibitory activity of flavonoids with a similar chemical structure in which myricetin was 40 times more effective than dihydromyricetin, which was explained by the results of molecular docking, where it was concluded that the different orientations in the active center of *α*-amylase may be an influencing factor on the inhibitory activity [48]. For the acidic phenol family represented by gallic acid, its effect was manifested at slightly higher concentrations of 0.23 mg/mL and 0.45 mg/mL, strengthening the hypothesis of the effect of the poly-hydroxyl group of the chemical formula and its effect on the inhibition rate. With such characteristics, many studies have shown and concentrated on these bioactive compounds as the compounds with important antidiabetic properties [49, 50]. A similar study conducted by Mehenni et al. agrees with our currently obtained results [10]. The study result of Foddai et al. revealed the presence of a great capacity of *Pistacia lentiscus* extract to chelate the enzyme as *α*-amylase [51].

### 3.5. Molecular Docking

**Figure 5:**
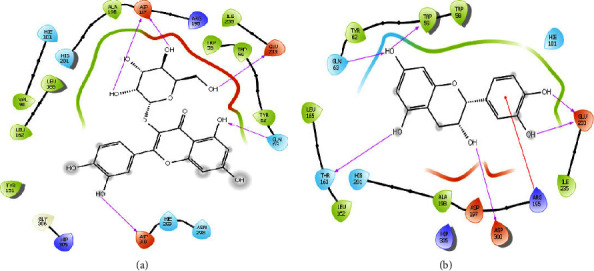
Ligand-protein interactions for (a) quercetin-3-glucoside and (b) catechin.

The study of the binding interactions of the six compounds with the *α*-amylase pocket was conducted to identify their inhibitory mechanism and to determine the active *α*-amylase site that allows hydrolysis comprising 3 large amino acids: GLU233, ASP300, and ASP197. The binding interactions of the compounds studied with respect to acarbose had sharing interactions, which showed that acarbose binds by five conventional hydrogen bonds with ASP300, ASP197, GLU233, HIE299, and ASP356. Quercetin-3-glucoside and acarbose have three similar hydrogen bonds with ASP300, ASP197, and GLU233, as shown in Figure 5. Besides, catechin and kaempferol have two hydrogen bonds similar to the active *α*-amylase site. Therefore, the majority of the studied compounds interact with the active *α*-amylase site via the hydrogen bonds, which leads to a good binding affinity against *α*-amylase, as shown in Table 4. Catechin, quercetin-3-glucoside, and kaempferol showed powerful *α*-amylase binding energy values than ellagic acid and gallic acid, with the following docking scores −7.631 kcal/mol, −6.818 kcal/mol, −6.204 kcal/mol, −5.320 kcal/mol, and −5.517 kcal/mol, respectively. Thus, the present result revealed that most of the compounds have minimal docking scores and remarkable binding affinity against *α*-amylase [52], which therefore could have provided the extract with the ability to inhibit *α*-amylase.

## 4. Conclusion

Identification of the polyphenolic compounds performed on the *Pistacia lentiscus* leaf extract was successfully achieved as indicated by the presence of 20 polyphenolic constituents. The total phenolic and flavonoid contents were quantified as 394.5 ± 0.08 (mg GAE/g DE) and 101.2 ± 0.095 (mg QE/g DE), respectively. The plant extract has been found to possess interesting antioxidant and antidiabetic properties. Future research should focus on elucidating the molecular mechanisms underlying the activities exhibited by the plant extracts and their active compounds. Additionally, conducting genotoxicity tests will enhance the reliability and validation of the obtained results. Finally, the molecular docking performed on the main phenolic compounds of extracts showed a marked binding affinity versus human pancreatic alpha-amylase, which allows it to explain the antidiabetic ability of the extract.

## Figures and Tables

**Table 1:** The elution process of the phenolic compound detection.

	Time (min)	*A* (%)	*B* (%)
1	0	95	5
2	10	60	40
3	15	50	50
4	20	40	60
5	25	0	100
6	30	0	100
7	35	95	5

**Table 2:** Retention times (RT) and *λ*_max_ of standard compounds by HPLC-DAD.

Compounds	RT (min)	Area (%)
Gallic acid	6.598	5.16
Catechin	11.879	22.37
Ellagic acid	20.645	2.13
Kaempferol	28.570	0.13
Quercetin 3-glucoside	30.801	4.44

**Table 3:** IC_50_ values for antioxidant and antidiabetic activities in hydroacetonic extract of *Pistacia lentiscus* leaves, along with total polyphenol and flavonoid contents.

Compounds	IC_50_ (mg/mL)			Total phenolic content (mg GAE/g dry extract)	Total flavonoid content (mg QE/g dry extract)
	*α*-Amylase inhibitory activity	Antioxidants			
		DPPH	BBC		
Extract	0.266 ± 0.01	0,0027 ± 0.002	0.128 ± 0.04	394.5 ± 0.08	101.2 ± 0.095
Acarbose	0.230 ± 0.02	—	—	—	—
Gallic acid	0.098 ± 0.001	—	—	—	—
Catechin	0.174 ± 0.001	—	—	—	—
Quercetin	0.058 ± 0.05	—	—	—	—
Ascorbic acid	—	0,0030 ± 0.003	—	—	—
Butylated hydroxyanisole (BHT)	—	—	0.004 ± 0.009	—	—

**Table 4:** The docking scores and the interacting amino acids of the identified phenolic compounds of *Pistacia lentiscus* extracts.

Compounds	Docking scores (kcal/mol)	Interacting amino acids	Types of bonds
Acarbose	−7.316	ASP300	H-bond
		HIE299	—
		ASP197	—
		GLU233	—
		ASP356	—

Catechin	−7.631	ARG195	Pi-cation
		GLU233	H-bond
		ASP300	—
		THR163	—
		GLN63	—
		TRP59	—

Quercetin-3-glucoside	−6.818	ASP300	H-bond
		ASP197	—
		GLU233	—
		GLN63	—

Kaempferol	−6.204	ASP197	H-bond
		GLU233	—
		TRP58	Pi-Pi stacking
		TRP59	—

Gallic acid	−5.517	ASP402	H-bond
		ARG398	—
		ARG252	—
		ARG252	Salt bridge

Ellagic acid	−5.320	ASP197	H-bond
		GLN63	—

## Data Availability

The data used in this study are available upon request from the corresponding author.
